# A Medical Witness on Medical Ethics and Things in General

**Published:** 1918-08-03

**Authors:** 


### A MEDICAL WITNESS ON MEDICAL ETHICS AND THINGS IN GENERAL.

The witness-box has both its own terrors and its own temptations, and the victim who surmounts the one is sometimes apt to be seduced by the other. Hence it is, we understand, an axiom in the art of cross-examination that a talkative witness should be encouraged rather than suppressed. Only give him rope enough and he will be sure to provide a noose for his own extinction. It was surely on this principle that a medical witness of high distinction was recently treated in a case heard in the King's Bench. He had, during his examination-in-chief, enlarged with much eloquence on the ethical value of the work and influence of the British Medical Association and was evidently quite at home in the rostrum and ready, therefore, for the temptation of the general proposition. "Have you never thought that this important work should be supported by Parliamentary sanction?" sarcastically asks the cross-examining counsel. "In ethical matters Acts of Parliament are useless," replies Sir John, a commonplace which has a certain measure of plausibility. But by this time the flood-gates are fully open, and there escapes the further proposition, "I distrust Parliament in most matters." Almost inevitably follows the question, "Would you then abolish Parliament?" and, prudence and caution having now fully evaporated under the opportunity for personal eloquence, there appears the reply, "I would abolish nine out of ten of the members." To this fatal exhibition was politely and gracefully conducted a witness whose chief purpose in the box was to exhibit the fashion in which a man of impartial and judicial mind looked from the outside on the work of the Association to whose credit he desired to speak. The wisdom of silence can hardly be practised in the witness-box. But the prudent person will carefully abstain from the other extreme. There lie dangers manifold, and experienced counsel well know how with perfect courtesy to lead the expansive witness to the entanglements.

There was one statement in Sir John Tweedy's evidence which demands a more serious word. He defended the ethical proceedings of the British Medical Association as supplying a want which was not otherwise met. Medical students, since the abolition of the apprenticeship system, he affirmed, have no ethical instruction; their training is purely technical, and thus they go out into the world quite unprepared to meet the social and personal difficulties of medical practice. If this is so, all we can say is that a more severe censure on the teachers in our medical schools could hardly be recorded. For at least five years these gentlemen have the students under the influence of their precepts and example, and if all that is done is to supply technical training the sooner the teachers give way to men of character and individuality the better. Sir John Tweedy, even allowing for the seduction of an attentive Court, speaks with some authority on the subject of medical teaching, seeing that he was for years himself a teacher and that he has been president of one of the surgical colleges. Doubtless, therefore, his censure has a wide application. But we are glad to know that it has not a universal application. On the contrary, there are medical schools where systematic efforts are made to equip the student with the sound principles upon which rest all professional relations, and by which, in the public interest, professional conduct is governed. Apart from general influences directed to this end there are the particular opportunities offered in the teaching of forensic medicine, and we will add that the teacher who neglects these is not alive to his duty and might with advantage be transferred to some other sphere of usefulness. There are no strange and mysterious doctrines to be expounded. Medical ethics contain no clause which needs a sanction outside the welfare of the patient and the public well-being.

